# Book Reviews

**Published:** 1894-03

**Authors:** 


### *BOOK REVIEWS.*

---

A SYSTEM OF GENITO-URINARY DISEASES, SYPHILOLOGY AND DERMATOLOGY. By various authors. Edited by Prince A. Morrow, A. M., M. D., Clinical Professor of Genito-Urinary Diseases, formerly Lecturer on Dermatology in the University of the City of New York, etc. With illustrations. In three volumes. Vol. II. Syphilology. New York, D. Appleton & Co., 1893.

The first volume of this valuable work has received mention in THE JOURNAL. In the present volume we find the different branches of the subject of syphilis treated by authors especially chosen for their fitness. Most of the names are familiar to readers of the literature of the subject, and their selection has been well made.

James Nevins Hyde, of Chicago, has the opening chapter on "History, Geographical Distribution, Evolution and General Pathological Anatomy of Syphilis."

John A. Fordyce, M. D., of New York, has a chapter on "The Etiology of Syphilis." He gives a history of the search for the specific micro-organism of the disease. This particular organism is not yet definitely isolated. The influence of pyogenic cocci in producing suppuration of syphilitic lesions is mentioned. Sources and vehicles of syphilitic contagion are then taken up by the writer. The production of syphilis in the lower animals has not yet been definitely accomplished.

Dr. L. Duncan Bulkley, of New York, writes the chapter on "Modes of Infection." He gives four. (1) Direct contact. (2) Mediate infection. (3) Hereditary transmission. (4) Maternal infection. In addition to venereal contact he mentions kissing, biting, sucking (as having the breast emptied by a professional breast-drawer), manual contact as from or to a patient in medical practice, "social infection" from other than sexual intercourse. Ordinary bodily contact is another method of acquiring the disease.

In the chapter on "Primary Syphilis," Dr. Edward Bennet Bronson describes the incubations, their length, the initial lesion, primary adenopathy, location of the primary lesions, the diagnosis, pathology and prognosis, and goes into the "theory of primary syphilis."

Dr. Jos. Zeisler, of Chicago, describes Constitutional Syphilis.

Dr. Prince A. Morrow, the editor, writes the chapter on the "Syphilodermata." He says they may occur in every form of lesion which is capable of being produced by the skin. "Special characteristics," the "regular evolution," "general symptomatology," and a *résumé* of all the peculiarities of the symptoms, their cause, etc., then the morbid anatomy, the prognosis, classification and a description of the different kinds of eruptions. Here, as elsewhere, are some excellent plates; in fact we have never seen better illustrations of the different eruptions.

Dr. W. R. Townsend describes "Bone Syphilis"; Dr. John Noland McKenzie "Syphilis of the Upper Air Passages"; Dr. W. T. Councilman, "Visceral Syphilis." There are some beautiful drawings of miscroscopical sections here, though the liver specimen does not show a reproduction of the appearance of the liver in the normal parts around the gummatous lesion.

Dr. J. B. Sachs describes "Syphilis of the Nervous System," giving some case histories. Dr. Charles Stedman Bull describes "Syphilis of the Eye and Its Appendages, both in Acquired and Hereditary Syphilis." "Syphilis of the Ear" is by Dr. J. Orne Green. F. R. Sturgis, M. D., treats of "Hereditary Syphilis" very fully. Herman S. Klotz writes on the "Diagnosis and Prognosis." J. William White, M. D., Boston, gives the "Treatment." Dr. S. T. Armstrong writes on "Syphilis in its Relation to Public Health." Dr. Edw. Martin on "Chancroid"; Tuttle on "Chancroid of Anus and Rectum." The book, as a whole, is eminently valuable.

M. B. H.

---

THE DISEASES OF THE NERVOUS SYSTEM. A Text-book for Physicians and Students. By Dr. Ludwig Hirt, Professor at the University of Breslau. Translated with permission of the author by August Hoch, M. D., and Frank R. Smith, M. D., Assistant Physician to the Johns Hopkins Hospital. Published by D. Appleton & Co., New York, 1893.

Dr. Hirt, in the original, has treated this subject in a manner quite original, and with a master hand. The whole work displays much investigation, great thought and earnest effort. His authorities are the best, and his own thoughts not to be surpassed. No work on the diseases of the nervous system deserves more favorable comment, and none will give more satisfaction. Prof. Hirt is honest to a fault, pessimism being the outcome. This, though sad, is nevertheless true, for many of the diseases of this wonderful system are progressive organic derangements, and in consequence are in a large degree hopeless as to cure. I think his, that is, Prof. Hirt's, advice to the practicing physician in his relationship to his patient most beautiful, if I may use the expression, and eminently honest, and if followed will do much to keep immaculate the noble calling of those practicing medicine. Dr. Hirt comes as near telling what is to be hoped for as any writer after whom I have read. The translators have done good and faithful work, and, having read the original, I take much pleasure in recommending the volume to the profession, assuring them that they will get fully the author's meaning and intent.

H. H.

---

AN AMERICAN TEXT-BOOK OF GYNECOLOGY. Edited by J. M. Baldy, M. D., Professor of Gynecology in the Philadelphia Polyclinic; Gynecologist to the Philadelphia Polyclinic, and the Pennsylvania Hospital, and Surgeon to the Gynecean Hospital. W. B. Saunders, Philadelphia.

America has the honor of placing gynecology in the high position which it holds to-day. The development has been so rapid that there has been no representative book on the subject until the American Text-Book series added this volume. It is the only complete work which brings the subject up to present date.

All discussion and every discarded idea is omitted, giving a concise, clear and complete treatise upon the subject, written by the representative teachers of gynecology in every section of the United States. It is so compiled that the text is uniform, and not a collection of monographs. It is a matter of regret that credit is not given to each author for the subject upon which he had written. One of the best features of the work are the illustrations, which give more anatomy and pathology than could be obtained from pages of description. The chapters on examination of the pelvic organs and the technique of gynecological operations are especially useful and practical. J. K. C.

---

NERVOUS EXHAUSTION (NEURASTHENIA). Its Symptoms, Nature, Sequences and Treatment. By Geo. M Beard, M. D., Fellow of the New York Academy of Medicine, Member of the American Neurological Association, etc. Edited with Notes and Additions by A. D. Rockwell, M. D., Professor of Electro-Therapeutics in the New York Post-Graduate Medical School, etc. Third Edition. Price, $2.75. E. B. Treat, 5 Cooper Union, New York.

It was Dr. Beard, the author of this volume, who first described the condition now known as nervous exhaustion. His first paper on this subject was given to the profession in 1868, based upon a study of thirty cases, but his views and conclusions met with little sympathy and attracted only the slightest attention. The author's work on the subject, now in its third edition, is the outcome of constant study over many years. Though particularly an American disease, neurasthenia is observed in Europe where Dr. Beard's treatise has been translated into several languages. The subject could not receive better treatment from any one than the learned gentleman who first described it and gave the best years of his life to its study. This is the most exhaustive discussion of the matter in the language, and must remain a classic as long as neurasthenia remains a disease. The present edition contains many notes and additions from Dr. A. D. Rockwell, for many years Dr. Beard's colleague in literary work. We take great pleasure in recommending a work of so great interest and value.

How to Use the Forceps. By Henry G. Landis, M. D., Professor of Obstetrics and Gynecology in Starling Medical College, Columbus, Ohio. Revised and Enlarged by Chas. H. Bushong, M. D., Assistant Gynecologist to Demilt Dispensary. New York. Price, $1.75. E. B. Treat, 5 Cooper Union, New York.

This book is a very intelligent discussion of the indications for the use of the forceps and of the right manner of using them. The right use of them demands a thorough knowledge of four things—the instrument itself, the maternal passages, the child's head, and the normal mechanism of labor. The book attempts to impart a correct knowledge of these essentials. The author believes in the judicious and timely use of the forceps. He does not advocate a hasty resort to instrumental delivery, but believes that lives are often lost, and the suffering of woman unnecessarily prolonged by a failure to deliver by forceps at the proper time. This book is an excellent one of its kind, and in every way worthy of careful study.

---

### Lippincott's Magazine for March, 1894.

The complete novel in the March number of *Lippincott's* is "A Desert Claim," by Mary E. Stickney. If is a charming tale of ranch life in Northern Colorado.

Gilbert Parker's serial, "The Trespasser," reaches its ninth chapter. "The Inmate of the Dungeon," by W. C. Morrow, is a story of uncommon power. Joel Chandler Harris in "The Late Mr. Watkins of Georgia; His Relation to Oriental Folk Lore," compares a curious legend of his own State with one of India.

In "A Prophet of the New Womanhood," Annie Nathan Meyer considers Henrik Ibsen from an unfamiliar point of view. Emma Henry Ferguson tells "More about Captain Reid," the Confederate blockade-runner.

John Gilmer Speed describes "The Training of the Saddle-Horse." Dr. Charles C. Abbott writes of "Bees and Buckwheat," and Charles McIlvaine of "The Evolution of Public Roads." In "Talks with the Trade," the subject of "Literary Mendicancy" is presented.

The poetry of the number is by Anna Robeson Brown and John James Meehan.

